# Postpartum depression and autoimmune disease: a bidirectional Mendelian randomization study

**DOI:** 10.3389/fpsyt.2024.1425623

**Published:** 2024-08-29

**Authors:** Wenlong Yu, Bingxue Su, Chaoqun Wang, Qing Xia, Yinxiang Sun

**Affiliations:** ^1^ School of Pharmacy, Zunyi Medical University, Zunyi, Guizhou, China; ^2^ Department of Pharmacy, Zhuhai People’s Hospital (Zhuhai Clinical Medical College of Jinan University), Zhuhai, Guangdong, China

**Keywords:** postpartum depression, autoimmune disease, Mendelian randomization, genetic cause, etiology

## Abstract

**Purpose:**

The rising prevalence of postpartum depression (PPD) is harmful to women and families. While there is a growing body of evidence suggesting an association between PPD and autoimmune diseases (ADs), the direction of causality remains uncertain. Therefore, Mendelian randomization (MR) study was employed to investigate the potential causal relationship between the two.

**Methods:**

This study utilized large-scale genome-wide association study genetic pooled data from two major databases: the IEU OpenGWAS project and the FinnGen databases. The causal analysis methods used inverse variance weighting (IVW). The weighted median, MR-Egger method, MR-PRESSO test, and the leave-one-out sensitivity test have been used to examine the results’ robustness, heterogeneity, and horizontal pleiotropy.

**Results:**

A total of 23 ADs were investigated in this study. In the IVW model, the MR study showed that PPD increased the risk of type 1 diabetes (OR , = 1.15 (1.05–1.26),p<0.01),Hashimoto’s thyroiditis((OR) = 1.21 (1.09–1.34),p<0.0001),encephalitis((OR) = 1.66 (1.06–2.60),p<0.05). Reverse analysis showed that ADs could not genetically PPD. There was no significant heterogeneity or horizontal pleiotropy bias in this result.

**Conclusion:**

Our study suggests that PPD is a risk factor for type 1 diabetes, Hashimoto’s thyroiditis, and encephalitis from a gene perspective, while ADs are not a risk factor for PPD. This finding may provide new insights into prevention and intervention strategies for ADs according to PPD patients.

## 1 Introduction

Postpartum depression (PPD), a prevalent major depressive following childbirth, affects 17.22% women worldwide with a higher prevalence in developing countries compared to developed countries (1). PPD is characterized by symptoms such as depression, emotional instability, feelings of guilt, loss of appetite, low self-esteem, and sleep disturbances, along with a 20% increase in suicidal ideation (2–4). Not only does PPD affect the women themselves, but may also increase the risk of depression in partners and mental retardation in children, increasing the economic burden on families (5–7). Life circumstances (8), social and psychological stress (9), postpartum grief (10), prenatal depression (11), lifestyle (12), vaginal delivery (13), hormonal changes (14), and marital or partner dissatisfaction (11) have been shown to be the common risk factors for PPD. Notably, the correlation between autoimmune diseases (ADs) and PPD has been underexplored in existing studies.

Recent research has highlighted the complex interplay between ADs and PPD. ADs are disease states caused by an immune response of the body’s immune system against its own components due to the fact that it is impossible to distinguish between self and non-self (15). A recent nationwide sibling comparison study demonstrated a bidirectional association between PPD and ADs (16). Although several observational studies have shown that women with ADs are at higher risk for postpartum depression, studies on MS are conflicting (17–21). Notably, preliminary evidence suggests a potential risk of subsequent ADs development in individuals with PPD (22, 23). Despite these insights, the genetic underpinnings of the relationship between PPD and ADs remain poorly understood, with a gap in research elucidating this aspect.

The objective of this study was to examine the causal relationship between PPD and autoimmune diseases (ADs) using bi-directional Mendelian randomization (MR) analysis. MR is a robust method that utilizes genetic variation as instrumental variables to establish causal relationships between risk factors and diseases (24). This method can play a critical role in addressing issues related to confounding factors and reverse causality in observational studies (25). Bi-directional MR analysis can effectively investigate the impact of ADs on the risk of postpartum depression (PPD), as well as the risk of ADs following PPD.

## 2 Methods

### 2.1 Study overview

**Figure 1:**
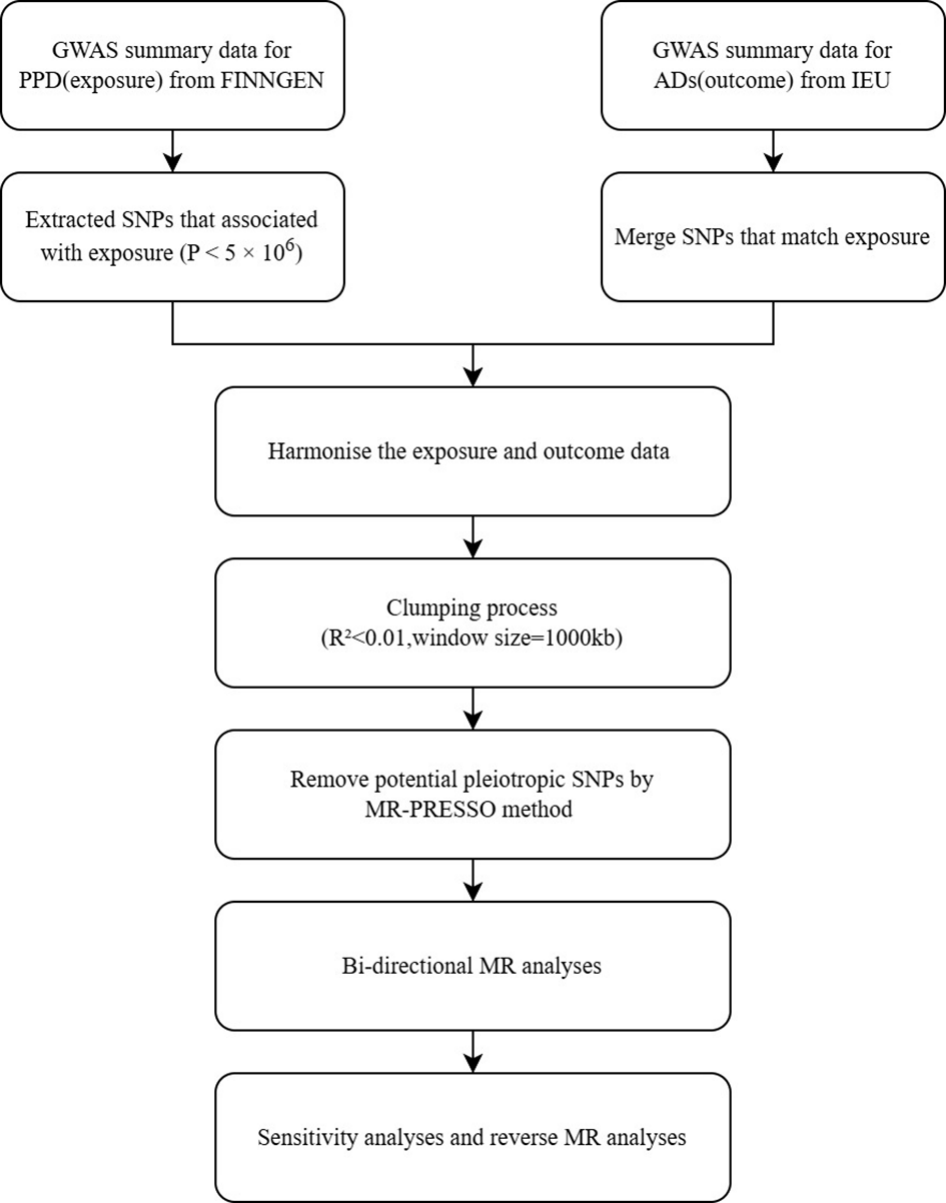
Study design and workflow.

In this study, we investigated the causal relationship between ADs and PPD using a bi-directional Mendelian randomization (MR) approach. We selected 23 subtypes of autoimmune disease diagnoses and obtained their Genome-Wide Association Study (GWAS) summary statistics data from publicly accessible databases. The initial research received ethical approval and informed consent. The study’s flow chart is depicted in **Figure 1**.

### 2.2 Data sources

Genome-wide association study (GWASs) summary statistics data for PPD from FinnGen R8, survey of 14,116 European women (prevalence 7.11%, mean age 41.03 years) (26). The diagnostic criteria for PPD are delivery status and International Classification of Diseases, Tenth Edition (ICD-10) codes F32, F33 and F53.0.

GWAS data for ADS were obtained from the IEU Open GWAS project (https://gwas.mrcieu.ac.uk/), which includes data from the UK Biobank (27), FINNGEN (26), and the International Multiple Sclerosis Genetics Consortium (28). The study includes the following ADs: Type 1 diabetes (29), Graves’ disease (30), Hashimoto thyroiditis (30), Rheumatoid arthritis (30), Ankylosing spondylitis (26), Giant cell arteritis with polymyalgia rheumatica (26), Polyarteritis nodosa and related conditions (26), Allergic purpura (26), Behcet’s disease (30), and Systemic lupus erythematosus (30), Psoriasis vulgaris (30), vitiligo (26), alopecia areata (26), idiopathic thrombocytopenic purpura (30), multiple sclerosis (28), myasthenia gravis (31), encephalitis (26), Guillain-Barre syndrome (26), ulcerative colitis (26), Crohn’s disease (32), coeliac disease (26), IgA nephropathy (30), and sarcoidosis (30). Detailed information on the ADs data is in **Supplementary Table 1**.

### 2.3 Instrumental variable selection

In magnetic resonance analyses of PPDs with ADs, the following three conditions were employed in order to select the optimal instrumental variable (IV) in order to ensure that the results were true and accurate: (I) the IV was closely related to PPDs, (II) the IV was not related to confounders and (III) the IV was not related to ADs (33). We selected SNPs that were significantly associated with PPD as IVs, choosing only those that were smaller than the genome-wide statistical significance threshold (5 × 10^-6)^. To ensure independence between IVs, SNPs with linkage disequilibrium were filtered using a clump window of 10,000 kb and r2 > 0.001. F-statistics were calculated for all independent variables (IV) to ensure that the F-statistics for the SNPs used in the analyses were all greater than 10. SNPs significantly associated with the results (p < 10^-8^) were also excluded. To prevent any distortion of strand orientation or allele coding, we removed palindromic SNPs (e.g. A/T or G/C alleles).

### 2.4 MR analysis

The inverse-variance weighted (IVW) MR method was applied as the primary method to identify potential associations between ADs and PPD (24). To evaluate the stability of the IVW results, we also used MR-PRESSO weighted median, heterogeneity test, MR-Egger regression heterogeneity test, Cochrane’s Q test, and weighted median (34, 35). The Cochrane Q test was used to assess the heterogeneity of the SNPs, and heterogeneity was present if P < 0.05 (36). Directed pleiotropy of genetic tools was tested using MR-Egger regression (37). To exclude SNPs whose abnormalities would affect our results, we also performed a leave-one-out sensitivity test (36). By analyzing the same trends in IVW and weighted median and MR-Egger analyses, the relationship between exposure and outcome was confirmed.

Analysis was performed using R 4.3.2 and the TwoSampleMR package.

## 3 Results

### 3.1 IVs selection

After rigorous screening, a total of 28 SNPs strongly associated with PPD (p<10^-6^) were used in this study. Detailed snps information can be found in **Supplementary Table 2**.

### 3.2 MR analysis of PPD for ADs

**Figure 2:**
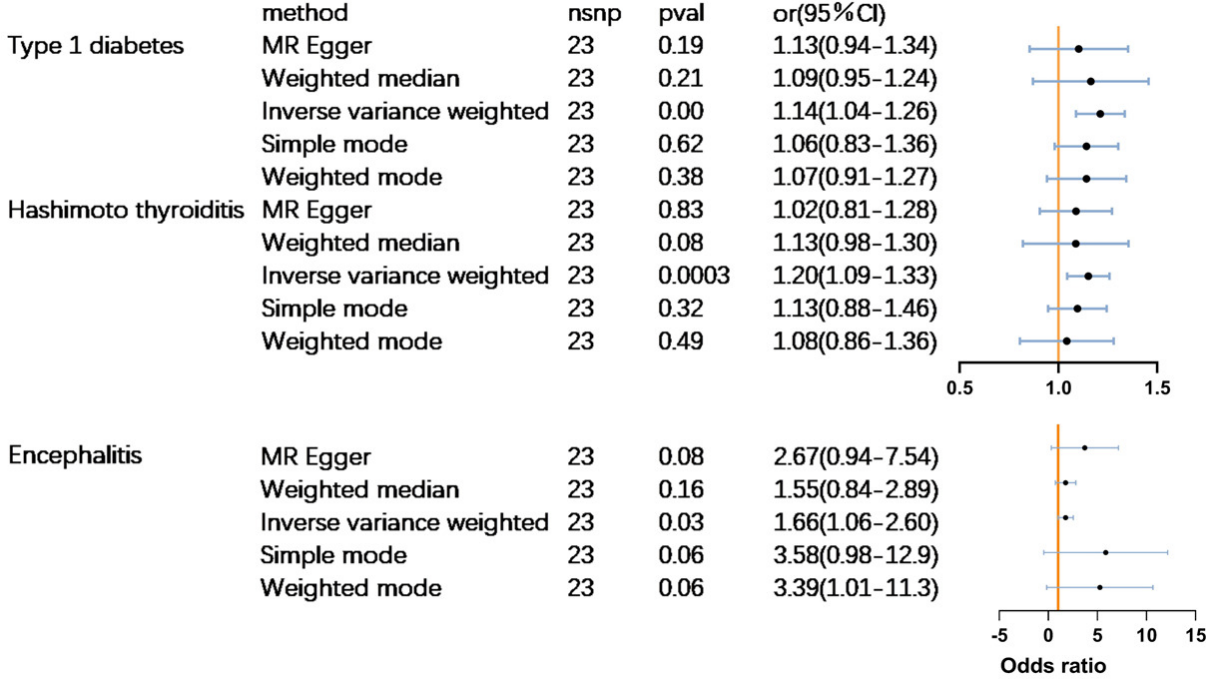
Main Mendelian randomisation results and forest plots.

MR analysis revealed that PPD had a significant causal relationship(p<0.05) with three out of the 23 autoimmune diseases. Women who suffer from PPD are at a higher risk of developing type 1 diabetes (odds ratio (OR) = 1.15 (1.05–1.26),p<0.01), Hashimoto’s thyroiditis [(OR) = 1.21 (1.09–1.34],p<0.0001), and encephalitis [(OR) = 1.66 (1.06–2.60),p<0.05] (**Figure 2**). No significant causal association was found between PPD and the other 20 subtypes of ADs. Complete results are available in **Supplementary Table 3**.

### 3.3 Sensitivity analysis of MR

**Figure 3:**
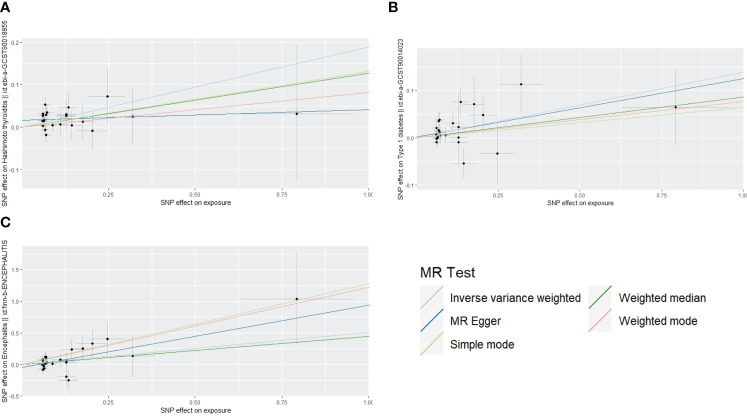
Scatterplot of main results.

The scatterplot shows that MR-Egger, weighted median, weighted mode, and simple mode results all follow the same trend as IVW:A for Hashimoto’s thyroiditis, B for type 1 diabetes, C for encephalitis (**Figure 3**). The test for heterogeneity was conducted using Cochrane’s Q-statistics, and no heterogeneity (p>0.05) was found for any of the three outcomes (**Table 1**). Tests of pleiotropy indicated no horizontal pleiotropy for type 1 diabetes (p=0.84), Hashimoto’s thyroiditis (p=0.13), and encephalitis (p=0.33).

**Table 1:** Heterogeneity testing using the Cochrane Q statistic.

outcome	method	Q	Q df	Q pval
Type 1 diabetes	MR Egger	23.43	21	0.32
	Inverse variance weighted	23.48	22	0.37
Hashimoto thyroiditis	MR Egger	15.20	21	0.81
	Inverse variance weighted	17.69	22	0.72
Encephalitis	MR Egger	20.11	21	0.51
	Inverse variance weighted	21.11	22	0.51

## 4 Discussion

In this study we further validated the causal relationship between PPD and ADs using MR in a European population. Our findings provide robust support for the involvement of PPD in the risk of Type 1 diabetes, Hashimoto’s thyroiditis, and Encephalitis. Sensitivity analyses also confirmed the reliability of our results, indicating that MR analyses of PPD are trustworthy. However, no association was observed between PPD and the remaining 20 ADs (p>0.05).

The association between PPDs and ADs is a topic of complexity and controversy. An observational study in Sweden found a bidirectional association between certain ADs (such as autoimmune thyroid disease, psoriasis, multiple sclerosis, ulcerative colitis, and celiac disease) and perinatal depression among unaffected sisters, independent of psychiatric comorbidity (16). However, previous studies have produced conflicting results (21, 38–41). Additionally, various studies have shown that inflammatory bowel disease, rheumatoid arthritis, systemic lupus erythematosus, multiple sclerosis, and psoriasis can increase the risk of PPD (18–20, 22, 40). However, the focus of most previous studies has been on investigating the risk of PPD following ADs, with limited research on the risk of subsequent ADs associated with PPD. A survey from Canada indicated that women with perinatal psychiatric disorders were at a higher risk of developing ADs, though not significantly different from women with non-perinatal psychiatric disorders (17). These findings were mainly based on observational studies that were unable to consider all potential mediators influencing the results, leading to controversies. Factors such as small sample sizes, confounding variables, reverse causation, and differences in study designs may contribute to the inconsistencies in the literature.

In this study, evidence was found suggesting a potential association between Postpartum Depression (PPD) and Type 1 diabetes, Hashimoto’s thyroiditis, and Encephalitis. Type 1 diabetes is a chronic autoimmune disease typically occurring in childhood, although the American Diabetes Association (ADA) has classified latent autoimmune diabetes in adults as T1DM as well (42, 43). This form of diabetes is thought to stem from a combination of genetic and environmental factors, presenting as heterogeneous at different stages (44). The progression of the disease may be exacerbated by psychological factors and obesity, leading to pancreatic beta cell exhaustion and autoimmune destruction (45). Moreover, latent autoimmune diabetes in adults and PPD are commonly linked to obesity, physical inactivity, and lifestyle factors (46, 47). Physiologically, PPD may be associated with type 1 diabetes through thalamic damage and HbA1c levels, indicating a potential pathway connecting the two conditions (48–50).

Hashimoto’s thyroiditis, an autoimmune thyroid disorder, is predominantly observed in women and results from a blend of genetic and environmental factors (51). Hormonal fluctuations during the perinatal phase and heightened stress in the postpartum period are believed to heighten the vulnerability of women with postpartum depression (PPD) to developing Hashimoto’s thyroiditis (52, 53). The occurrence of pregnancy leads to notable modifications in thyroid function, with variations in hormone levels such as human chorionic gonadotropin, estrogen, and progesterone being associated with the initiation and progression of Hashimoto’s thyroiditis (22, 52). It is crucial to closely monitor thyroxine levels in women with PPD to forestall autoimmune thyroid disease. Research findings suggest that individuals with chronic mental disorders have a low likelihood of acquiring autoimmune encephalitis (54). There is a proposition that individuals enduring postpartum psychosis may exhibit higher susceptibility to encephalitis (55). Our study’s outcomes reveal that postpartum depression is linked to an elevated risk of encephalitis, as indicated by a higher odds ratio (OR=1.6). Our results suggest that patients with PPD have an increased risk of type 1 diabetes, Hashimoto’s thyroiditis, and encephalitis associated with their genetic susceptibility.

The use of genetic variation consistent with Mendel’s law of random assignment as an instrumental variable allows for the exclusion of confounding factors in MR analysis (24). This method addresses the issue of reduced confidence in previous observational studies on the relationship between PPD and ADs, which was attributed to the presence of confounding factors and reverse causality that were difficult to avoid. Furthermore, since these single nucleotide polymorphisms (SNPs) are strongly associated with disease and exist before the onset of disease, reverse causation is no longer a concern (56). The GWAS summary data selected for this study were derived from research with large sample sizes, enhancing the reliability of the results.

Several limitations need to be considered in our study. Firstly, the analysis was confined to GWAS studies conducted in Europe, thus it would be advantageous to incorporate data from other regions. Moreover, the study population consisted solely of females; however, it is worth mentioning that most studies utilizing GWAS data for autoimmune diseases did not differentiate between genders.

This is the first study to employ MR to investigate the potential causal relationship between ADs and PPD. The findings indicate that PPD is associated with an increased risk of developing Type 1 diabetes, Hashimoto’s thyroiditis, and Encephalitis. Further experimental and mechanistic studies are required to validate the results obtained.

## Data Availability

The original contributions presented in the study are included in the article/**Supplementary Material**. Further inquiries can be directed to the corresponding authors.
